# Prevalence, frequency, and disability of migraine headaches and tension headaches among the general population in the Eastern Region of Saudi Arabia

**DOI:** 10.25122/jml-2022-0176

**Published:** 2022-11

**Authors:** Mohammed AlBarqi, Mohammed AlDablan, Abdulelah AlBahr, Mohammed AlAmer, Abdulrahman AlNaim, Abdullah AlNaim, Abdullah Almaqhawi

**Affiliations:** 1 Department of Family Medicine and Community, College of Medicine, King Faisal University, AlAhsa, Saudi Arabia; 2 College of Medicine, King Faisal University, AlAhsa, Saudi Arabia

**Keywords:** headaches, risk factors, prevalence, Saudi Arabia

## Abstract

A tension-type headache (TTH) is a recurrent headache that is mild to moderate in intensity, unlike migraine (MH), which is accompanied by crippling effects of nausea, vomiting, photophobia, or phonophobia. TTH is more frequent than migraine, but it is less likely to cause severe pain and physical dysfunction. TTHs account for more lost workdays due to their prevalence. The study aimed to evaluate the prevalence, frequency, and disability of MHs and TTHs among the general population in the Eastern Region of Saudi Arabia. A cross-sectional study was employed using a validated questionnaire. The questionnaire implemented the HARDSHIP algorithm to diagnose MH and TTH and questions to correlate their prevalence to socio-demographic data, measurement of the level of disability, headache management, and treatment effectiveness using the chi-square test. The study reviewed 877 subjects (46.6% males *vs*. 53.4% females). 52.9% of the participants experienced headaches during the previous three months, and 35.6% experienced headaches recently. The most common type of headache was a probable MH (32.1%), followed by a TTH (26.9%), probable TTH (19.5%), and MH (15%). It was reported that some disability was attributed to 47% of MHs and 26% of TTHs. The most commonly used headache medication was paracetamol (53.5%). This study revealed that TTH and MH are common illnesses in Saudi Arabia's Eastern Region. TTH and MH are correlated with significant individual and social burdens, particularly for MH. Headache sufferers mostly manage their headaches using conventional over-the-counter methods.

## INTRODUCTION

Headache is one of the most prevalent complaints of patients seen in internal medicine and adult neurology clinics [1]. Although it is among the top 10 complaints in outpatient medical care, we still do not fully grasp the epidemiology of headache problems. Most recurring headache cases are brought on by benign, primary, chronic headache diseases, including tension-type headache (TTH) and migraine (MH). Less commonly, other underlying illnesses such as infections, cerebral hemorrhages, and brain lesions might cause headaches [2].

Migraine is marked by symptoms of nausea, vomiting, phonophobia, and photophobia and last four to 72 hours. They are unilateral, have a pulsating quality, moderate to severe intensity, and are exacerbated by physical activity. Aura signs can occur before a migraine [3]. Emotional stress, poor or prolonged sleep, odors, skipping a meal, and menstruation are all potential triggers of migraine attacks [4]. Active MHs are estimated to affect 26.97% of people in Saudi Arabia, 14% of people in Europe, and 11% of the global population [5, 6]. Migraine incidents increase after puberty, and women are more likely than men to suffer from them [7]. According to some research, the prevalence of MHs is rising due to unidentified causes [8].

Headaches and MHs are significant contributors to the burden of treatment in primary care services due to their high prevalence rates [7]. Headache disorders are the second leading cause of years lived with disability worldwide, according to the burden of disease research [9]. MH can be so debilitating that they necessitate repeated visits to outpatient clinics and emergency departments, resulting in substantial clinical and financial costs. In 2009, headaches were one of the top five reasons for emergency room admissions and one of the top 20 reasons for outpatient visits in the United States (US) [10]. Headaches, especially MHs, put a considerable financial and social strain on sufferers as well as society as a whole due to decreased quality of life, lost productivity, and the use of healthcare services [11]. In the US, the annual burden of MHs on employers is estimated to be around $13 billion, with annual treatment expenses above $1 billion [12]. Individuals suffering from MHs are more likely to experience underlying medical problems, chronic pain conditions, ischemic stroke, sleep disruption, depression, anxiety, and a rise in stress [13]. Despite the fact that MH is a serious disability, many individuals who suffer from it do not pursue medical treatment, and MHs are, therefore, significantly underreported and undertreated [14].

Tension-type headaches range widely from occasional short-term periods of discomfort to regular, long-term, or persistent ones in frequency, duration, or intensity [15]. TTH could coexist with MH often, but distinguishing between a TTH and milder types of MH without aura can be difficult [16]. A TTH is a recurrent headache that is mild to moderate in intensity and not accompanied by the crippling MH effects of nausea, vomiting, photophobia, or phonophobia [11]. In addition, a TTH is more frequent than MH, with a prevalence ranging from 30% to 70%, but it is less likely to cause severe pain and physical dysfunction. While MH sufferers are more likely to miss work, TTHs account for more missing workdays due to their prevalence [17]. A study in Denmark states that only 16% of people with TTH have contacted their general practitioner because of their TTH, compared to 56% of MH sufferers [18]. The cost of treating a TTH is twice as high as treating MHs, considering the higher incidence of TTHs [19].

To our knowledge, no study in the Eastern Region of Saudi Arabia focused on the disability caused by MHs and TTHs. To reduce the burden of headaches in the community and reinforce efforts to improve the patient's quality of life, it is critical to understand its distribution, related sociodemographic and clinical characteristics, and pathogenic processes. Identifying the underlying factors might result in enhanced preventative efforts and the early identification of vulnerable groups. We aimed to evaluate the prevalence, frequency, and disability of MHs and TTHs among the general population in the Eastern Region of Saudi Arabia.

## MATERIAL AND METHODS

A cross-sectional questionnaire-based study was conducted using a previously validated questionnaire adapted and distributed electronically through social media platforms to all adult headache sufferers from the Eastern Province of Saudi Arabia. The questionnaire included several questions, including socio-demographic data, headache-type diagnostic algorithms, measurement of the level of disability, headache management strategies, and their effectiveness. The study was conducted between August 2021 and March 2022. Participants were recruited through an online survey from the eastern region. Participants who refused or were below 18 or non-Arabic speakers were excluded.

Regarding the implemented diagnostic algorithm, the HARDSHIP algorithm [20] was used to classify episodic headaches in hierarchical sequence: first definite MH, then definite TTH, then probable MH, and finally, probable TTH. Cases falling into none of these categories were “undetermined”. During subsequent analysis, MH and probable MH were considered as one category in further discussion. In addition, TTH and probable TTH were considered as one category. Medication overuse headaches (MOH), which require direct clinical encounters with patients, were excluded.

According to the Saudi Arabia General Authority for Statistics, the latest statistics of the population of Eastern Province above 18 years of age are estimated at 2,356,197 [21]. Therefore, 385 participants recruited from Saudi Arabia's general population were considered the minimum sample size required for this study, with a 95% confidence interval and a 5% margin of error [22]. The data were analyzed using the Statistical Packages for Social Sciences (SPSS) version 26 (Armonk, NY: IBM Corp). Categorical variables were presented using numbers and percentages. The relationship between the MH and TTH overall prevalence was correlated to sociodemographics and patients' control over the conditions using the chi-square test. A p-value cut-off point of 0.05 at 95% CI was used to determine the statistical significance.

## RESULTS

**Figure 1:**
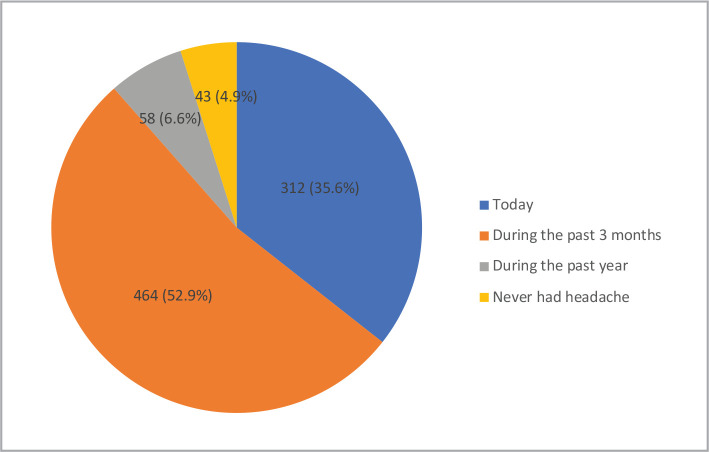
Last time participants experienced a headache.

**Figure 2:**
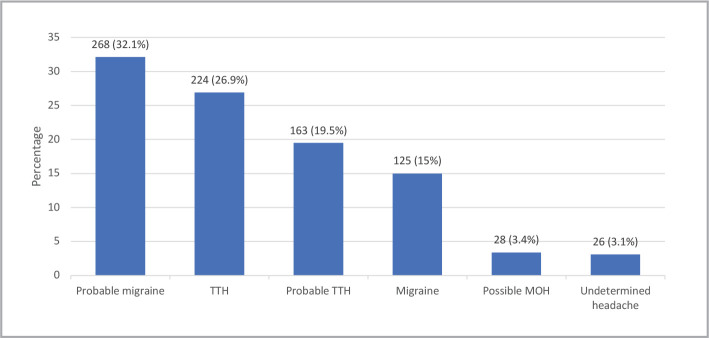
Type of headache.

This study contacted 877 subjects and excluded 43 individuals who had never experienced a headache, as shown in Figure 1. Furthermore, after analyzing the data through the HARDSHIP algorithms, we excluded the other unstudied types of headaches (possible MOH=28 subjects, undetermined headache=26 subjects), as illustrated in Figure 2. As seen in Table 1, the most common age group was 18–29 years (38.1%), nearly 60% were married, and a half (50.1%) were employed. Participants who earned less than 5,000 SAR per month constituted 42.4%, while others had monthly earnings of 10,001–15,000 SAR per month (21.3%) or 5,000–10,000 SAR per month (20.5%).

**Table 1:** Sociodemographic characteristics of the participants (n=877).

Study variables	N (%)
**Age in years**	
18–29 years	334 (38.1%)
30–39 years	228 (26.0%)
40–49 years	160 (18.2%)
≥50 years	155 (17.7%)
**Gender**	
Male	409 (46.6%)
Female	468 (53.4%)
**Marital status**	
Single	309 (35.2%)
Married	525 (59.9%)
Divorced	22 (02.5%)
Widowed	21 (02.4%)
**Occupational status**	
Employed	439 (50.1%)
Unemployed	117 (13.3%)
Retired	90 (10.3%)
Student	231 (26.3%)
**Monthly income (SAR)**	
<5,000	372 (42.4%)
5,000–10,000	180 (20.5%)
10,001–15,000	187 (21.3%)
>15,000	138 (15.7%)

Figure 1 shows the last time the participants experienced a headache. 52.9% of the participants experienced headaches during the previous three months, and 35.6% experienced headaches recently.

The prevalence of the type of headache is illustrated in Figure 2. The most common type of headache was a probable MH (32.1%), followed by a TTH (26.9%), probable TTH (19.5%), and MH (15%). In this study, MH and probable MH were considered as one category in further discussion. In addition, TTH and probable TTH were also considered as one category.

We then asked diagnostic questions to participants who experienced headaches (n=780). Following the results, we observed that around two-thirds (66.3%) of the participants experienced a headache for less than seven days during the previous month, which lasted between 30 minutes and four hours (53.6%). Furthermore, moderate headache was experienced by 58.5% of respondents, with pressure being the most common nature of headaches (50.5%). 66.7% reported that the headache was localized in one area, while 34.9% reported that the headache increased with exercise. Incidentally, the most common symptom reported was phonophobia (22.6%), followed by photophobia (13.6%), while 35.3% experienced both symptoms. In addition, 42.7% took medication for less than five days, and 48.6% did not take any medication at all (Table 2).

**Table 2:** Responses to diagnostic questions by participants who experienced headaches (n=780).

Statement	N (%)
**How often did you have headaches during the past month?**	
<7 days	517 (66.3%)
7–15 days	172 (22.1%)
>15 days	91 (11.7%)
**How long does a headache episode last?**	
Less than an hour	106 (13.6%)
Between 30 mins to 4 hours	418 (53.6%)
Between 4 hours to 3 days	208 (26.7%)
Between 3 days to 7 days	30 (03.8%)
More than 7 days	18 (02.3%)
**How severe is the headache pain generally?**	
Mild	177 (22.7%)
Moderate	456 (58.5%)
Severe	147 (18.8%)
**How would you describe the nature of the headache?**	
Pulsing	386 (49.5%)
Pressure	394 (50.5%)
**Is the headache localized in one area?**	
Yes	520 (66.7%)
No	260 (33.3%)
**Does the headache increase by exercise (i.e. walking)?**	
Yes	347 (44.5%)
No	433 (55.5%)
**Do you feel nausea or vomiting when you have a headache?**	
Yes	272 (34.9%)
No	508 (65.1%)
**Do you have any of these symptoms?**	
None	223 (28.6%)
Annoyed by light (photophobia)	106 (13.6%)
Annoyed by voices (phonophobia)	176 (22.6%)
Both	275 (35.3%)
**How many days do you take medicine?**	
<5 days	333 (42.7%)
Between 5 to 10 days	47 (06.0%)
More than 10 days	21 (02.7%)
Don't take any medication	379 (48.6%)

The disability questions among participants who experienced headaches are given in Table 3. It revealed that 38.1% of respondents indicated that the severity of headaches did not affect their daily lives. 56.3% of TTH patients could do everything normally; on the other hand, 36.4% of MH sufferers could only do half of what they needed to do. The significance of respondents who could do things normally at work or school despite their headache (p<0.001) was higher with TTH compared to MH, 66.7% of whom expressed that the headache did not affect their presence at work or school. With regard to the sub-type headache, 80.9% of TTH said it did not affect their presence at job or school, while 52.7% of those with MH said it did not affect their job or school. It is notable that those with MH missed a whole day in their work or school (p<0.001) in comparison to TTHs. However, 36.2% of them completed only half of their daily activities at either work or school as a result of a headache. Moreover, only 16.0% with MHs could do everything, while 34.1% of TTH could do all of their required activities. This can be related to the fact that respondents who reported that they could finish less than half of their daily activities due to headaches had an increased risk of MHs (p<0.001). Similarly, at least 79.7% of the respondents' work was affected by headaches for less than seven days during the previous three months, which was more pronounced in TTHs (86.3%). In addition, those with less than seven days of headaches during the previous three months (p<0.001) had an increased risk of having TTHs. Meanwhile, only 15.8% of the participants with MHs reported that they were fully understood by their colleagues and their supervisors, while 26.1% with TTHs reported that they were fully understood (p=0.002).

**Table 3:** The responses to disability questions according to the type of headache (n=780).

Statement	Overall N (%) (n=780)	TTH N (%) (n=387)	MH N (%) (n=393)	P-value ^§^
**How severely does the headache affect your daily life/work/school?**				
I do everything normally	297 (38.1%)	218 (56.3%)	79 (20.1%)	<0.001 **
I do half of what I need to do	200 (25.6%)	90 (23.3%)	110 (28.0%)	
I do less than half of what I need to do	214 (27.4%)	71 (18.3%)	143 (36.4%)	
I can't work at all	69 (08.8%)	08 (02.1%)	61 (15.5%)	
**Does the headache affect your presence at your job/school?**				
No	520 (66.7%)	313 (80.9%)	207 (52.7%)	<0.001 **
I go in late	79 (10.1%)	35 (09.0%)	44 (11.2%)	
I miss some of the day	114 (14.6%)	32 (08.3%)	82 (20.9%)	
I miss the whole day	67 (08.6%)	07 (01.8%)	60 (15.3%)	
**How do you complete your daily activities at work/school when you have a headache?**				
Nothing	50 (06.4%)	11 (02.8%)	39 (09.9%)	<0.001 **
Less than a half	282 (36.2%)	95 (24.5%)	187 (47.6%)	
More than a half	253 (32.4%)	149 (38.5%)	104 (26.5%)	
Everything	195 (25.0%)	132 (34.1%)	63 (16.0%)	
**How many days has the headache affected your work during the last three months?**				
<7 days	622 (79.7%)	334 (86.3%)	288 (73.3%)	<0.001 **
Between 7 days to 15 days	98 (12.6%)	37 (09.6%)	61 (15.5%)	
More than 15 days	34 (04.4%)	07 (01.8%)	27 (06.9%)	
More than a month	26 (03.3%)	09 (02.3%)	17 (04.3%)	
**Do you feel that your colleagues and your supervisor understand your headache issues?**				
They don't understand	215 (27.6%)	91 (23.5%)	124 (31.6%)	0.002 **
Understand a little	290 (37.2%)	143 (37.0%)	147 (37.4%)	
Understand a lot	112 (14.4%)	52 (13.4%)	60 (15.3%)	
Fully understand	163 (20.9%)	101 (26.1%)	62 (15.8%)	

§ – P-value has been calculated using the chi-square test; ** – Significant at p<0.05 level.

As shown in Table 4, the most commonly used medication for headaches was paracetamol (53.5%). On the other hand, 38.3% expressed that the medication significantly decreased the headache duration, 34.6% of whom were MH sufferers who experienced a minimum reduction in duration due to the effects of medications in comparison to those with TTHs (23.8%). Accordingly, 30.0% of the respondents reported that their headaches could not be controlled, and 36.1% of those with MHs said they could not control them in comparison to 23.8% with TTHs. In addition, the number of respondents who reported that they can control headaches a little (p<0.001) was significantly higher in the MH group. The prevalence of respondents who reported that they felt anxious, even during headache-free days, was 33.6%, with 43.8% of those suffering MH feeling anxious even during headache-free days, compared to 23.3% with TTHs. Moreover, the number of people who felt anxious during headache-free days (p<0.001) was significantly higher in the MH group, while the prevalence of respondents who could not do anything in fear of a headache was 20.8%, 30.0% of whom had MHs compared to 11.4% with TTHs. In addition, there was a significant relationship between those who indicated that they could not do anything during headache-free days in fear of a headache (p<0.001) being less in the TTH group.

**Table 4:** Treatment and post-headache characteristics according to the type of headache (n=780).

Statement	Overall N (%) (n=780)	TTH N (%) (n=387)	MH N (%) (n=393)	P-value ^§^
**If you are taking any medications for your headache, please specify**				
I don't take any	201 (25.8%)	115 (29.7%)	86 (21.9%)	<0.001 **
Paracetamol	417 (53.5%)	215 (55.6%)	202 (51.4%)	
Paracetamol + Ibuprofen	32 (04.1%)	05 (01.3%)	27 (06.9%)	
Aspirin	25 (03.2%)	13 (03.4%)	12 (03.1%)	
Ibuprofen	21 (02.7%)	07 (01.8%)	14 (03.6%)	
Paracetamol + Aspirin	15 (01.9%)	05 (01.3%)	10 (02.5%)	
Triptan	10 (01.3%)	0	10 (02.5%)	
Ibuprofen + Triptan	05 (0.60%)	0	05 (01.3%)	
Others	54 (06.9%)	27 (07.0%)	27 (06.9%)	
**Does the medication affect the headache duration?**				
I don't take medication	201 (25.8%)	115 (29.7%)	86 (21.9%)	0.004 **
Reduces the duration a lot	299 (38.3%)	151 (39.0%)	148 (37.7%)	
Reduces the duration minimally	228 (29.2%)	92 (23.8%)	136 (34.6%)	
Has no effect	52 (06.7%)	29 (07.5%)	23 (05.9%)	
**How do you view your control of your headache?**				
I don't control it	234 (30.0%)	92 (23.8%)	142 (36.1%)	<0.001 **
I control it a little	369 (47.3%)	174 (45.0%)	195 (49.6%)	
I control it a lot	111 (14.2%)	71 (18.3%)	40 (10.2%)	
I fully control it	66 (08.5%)	50 (12.9%)	16 (04.1%)	
**On headache-free days, do you feel anxious about your headaches?**				
Yes	262 (33.6%)	90 (23.3%)	172 (43.8%)	<0.001 **
No	518 (66.4%)	297 (76.7%)	221 (56.2%)	
**On headache-free days, do you find yourself unable to do anything fearing the headache?**				
Yes	162 (20.8%)	44 (11.4%)	118 (30.0%)	<0.001 **
No	618 (79.2%)	343 (88.6%)	275 (70.0%)	

§ – P-value has been calculated using the chi-square test; ** – Significant at p<0.05 level.

When measuring the relationship between the type of headache and the socio-demographic characteristics of the participants, it was found that being married (p<0.001), being employed (p<0.001), and having a headache for the previous three months (p<0.001) was more significant for having TTHs, while the age group of 18–29 years (p<0.001), females (p<0.001), and those with less than 5,000 monthly earnings (p<0.001) were significant for MHs (Table 5).

**Table 5:** Relationship between the type of headache and the socio-demographic characteristics of the participants (n=780).

Factors	TTH N (%) (n=387)	MH N (%) (n=393)	P-value ^§^
**Age in years**			
18–29 years	126 (32.6%)	174 (44.3%)	<0.001 **
30–39 years	98 (25.3%)	114 (29.0%)	
40–49 years	87 (22.5%)	59 (15.0%)	
≥50 years	76 (19.6%)	46 (11.7%)	
**Gender**			
Male	229 (59.2%)	135 (34.4%)	<0.001 **
Female	158 (40.8%)	258 (65.6%)	
**Marital status**			
Unmarried	127 (32.8%)	184 (46.8%)	<0.001 **
Married	260 (67.2%)	209 (53.2%)	
**Occupational status**			
Employed	224 (57.9%)	174 (44.3%)	<0.001 **
Unemployed	82 (21.2%)	95 (24.2%)	
Student	81 (20.9%)	124 (31.6%)	
**Monthly income (SAR)**			
<5,000	138 (35.7%)	201 (51.1%)	<0.001 **
5,000–10,000	83 (21.4%)	78 (19.8%)	
10,001–15,000	92 (23.8%)	75 (19.1%)	
>15,000	74 (19.1%)	39 (09.9%)	
**The last time having a headache**			
Today	115 (29.7%)	177 (45.0%)	<0.001 **
During the past 3 months	240 (62.0%)	194 (49.4%)	
During the past year	32 (08.3%)	22 (05.6%)	

§ – P-value has been calculated using the chi-square test; ** – Significant at p<0.05 level.

## DISCUSSION

The present study investigated the prevalence and disability of MH and TTH among the general population in the Eastern Region of Saudi Arabia. The prevalence of MHs in this study was 15%. This is consistent with the paper by AlQarni et al. [23]. Based on their accounts, the prevalence of MHs among adults living in the Aseer region of Saudi Arabia was 12.3%. For university students, the prevalence of MHs was in agreement with our results, with studies indicating that the prevalence of MHs among medical students was 15.4% and 17.9%, respectively [24, 25]. Other publications that have reported MH prevalence were within our range, including a study published in Nigeria [26], Brazil [27], Italy [28], and Turkey [29]. On the other hand, a study conducted in Taif, Saudi Arabia [30] detected a higher MH prevalence among female Saudi students at Taif University, at 32.5%, which was in accordance with the paper of Kandil et al. [31]. We further noted that the prevalence of probable MHs was higher in our population, accounting for 32.1%. This prevalence rate is higher than in other studies. For example, in Pakistan [24], the prevalence of probable MHs was 15.4%, while in Brazil [27], the prevalence was 8%, whereas, in Egypt, it was 12.5%. We have recorded that the prevalence of probable MH was the highest in our study, and no other study has recorded the same result.

The prevalence of TTH in this study (26.9%) lies within the range of studies, as reported in Taif, Saudi Arabia [30], and in Southeast Nigeria [26]. The highest prevalence rate of TTH was reported by Prencipe et al. [28], with a prevalence of 44.5%, which was surveyed among three villages in central Italy. Some papers have reported a lower prevalence rate. For instance, in a study conducted by Falavigna et al. [27], the prevalence of TTH was 12.8%, while in Kandil et al. [31], the prevalence of TTH was 13.6%. In addition, Ertas and colleagues [29] reported the lowest 1-year prevalence rate of 5.1%. The probable TTH prevalence among our population was 19.5%, which was in agreement with the study conducted among university students [24, 27]. However, in Turkey [29], the prevalence of probable TTH was lower, at 9.5%.

A younger age group (18–29 years), female respondents, and a low monthly income among participants demonstrated a significantly higher probability of having an MH. These findings are almost in agreement with the paper of Constantinides et al. [32], who found that MH patients had a significantly lower age at headache onset and frequency, higher mean visual analogue scale (VAS), and greater maximum duration of headache episodes compared to TTH patients. Similarly, we observed that the prevalence of TTH was significantly higher among working participants and those who had had a headache during the previous three months. In Egypt [25], researchers have observed a relationship between the severity of TTH and some of the demographic characteristics of the respondents. The study showed that the severity of TTH was associated with age, specifically after the age of 40, while prolonged TTH was found to be five-fold in unmarried individuals and two-fold in a large number of children.

In our further review, compared to TTH subjects, MH subjects described that 15.5% could not do their work at all, which represents a tremendous disability, while 47.6% of the MH sufferers stated that they could only finish less than half of their daily activities at work or school. This is consistent with the paper of Edmeads et al. [33], who reported that some disability was attributed to 47% of reported MHs and 26% of reported TTHs. Similarly, 15.3% of MH subjects missed the whole day either at school or at work. However, in America, they revealed that around 31% of all MH missed at least one day of work or school due to headaches [34]. Meanwhile, TTH subjects said that headaches affected them at work for less than seven days during the previous three months. In a paper conducted by Radtke et al. [35], MH was more severely impacted by headaches than non-migraine headache sufferers, as demonstrated by a higher number of headache days.

In the present study, compared to MH subjects, 56.3% of TTH subjects could do everything normally in their daily lives, and 80.9% expressed that the TTH did not affect them at all while they were at work or school. In addition, 86.3% of the TTH subjects stated that headaches affected them at work for a maximum of seven days during the previous three months, while 37% of the TTH subjects stated that their colleagues and supervisors understand them just a little. Incidentally, compared to TTH, 47.6% of MH sufferers stated that they were able to finish less than half of their daily activities at work or at school. In Egypt, however [25], they revealed that headaches increased with daily activities, with similar findings in Southern Brazil [27], where the pain worsened with the students' daily activities.

Paracetamol was the common choice of analgesic for the treatment of headaches, with 38.3% of the subjects stating that the medication reduced the duration a lot, and 42.7% indicated that they usually took medication for less than five days. Several studies have also indicated paracetamol is the chief medication for headaches [25, 29–31]. Oraby and associates [25] further stated that the self-prescription of medications for MHs was practiced by 58.4%, while 25.7% used doctor's prescriptions. In previous publications, paracetamol was regarded as the most popular due to its low price, safety, and less gastrointestinal tract (GIT) adverse effects. In addition, it can be purchased over the counter at any convenience or pharmacy store [36, 37].

## CONCLUSION

This study revealed that TTH and MH are common illnesses in Saudi Arabia's Eastern Region, accounting for 26.9% and 15%, respectively, and that they are correlated with significant individual and social burdens, particularly in MH sufferers. MH is a huge burden on the healthcare sector, as it causes more severe symptoms and more frequent attacks, which impose more disability on the patient. In addition, headache sufferers tend to manage their headaches using over-the-counter conventional methods, with less than 9% fully controlling their headaches.

To reduce the burden on the healthcare sector imposed by TTHs and MHs, we recommend raising awareness among physicians and patients to identify each type of headache and treat it accurately through correct and timely counseling and medication.

Further study is needed in the future to minimize errors and direct patients toward ways to lower the incidence of disability as much as possible. Per limitations, this study selected the population as general Eastern Province citizens, which could be specified in further studies to classify the Eastern Province population according to the city of residence.

## Data Availability

Further data is available from the corresponding author on reasonable request.
